# SGLT2 Inhibitor Might Prevent Atrial Fibrillation Related to Metabolic Syndrome via TNF‐α Signaling Pathway: A Bioinformatic Study

**DOI:** 10.1002/joa3.70197

**Published:** 2025-10-08

**Authors:** Ardian Rizal, Mohammad Saifur Rohman, Adhika Prastya, Fatchiyah Fatchiyah, Hidayat Sujuti, Ahmad Rudianto, Seskoati Prayitnaningsih

**Affiliations:** ^1^ Doctoral Program in Medical Science, Faculty of Medicine Universitas Brawijaya Malang East, Java Indonesia; ^2^ Department of Cardiology and Vascular Medicine, Faculty of Medicine Universitas Brawijaya/Dr. Saiful Anwar General Hospital Malang Indonesia; ^3^ Research Center of Smart Molecule of Natural Resource Universitas Brawijaya Malang Indonesia; ^4^ Departement of Ophthalmology, Faculty of Medicine Universitas Brawijaya/Dr. Saiful Anwar General Hospital Malang Malang East Java Indonesia; ^5^ Department of Internal Medicine, Faculty of Medicine Universitas Brawijaya/Dr. Saiful Anwar General Hospital Malang East Java Indonesia

**Keywords:** atrial fibrillation, metabolic syndrome, network pharmacology, sodium‐glucose transporter 2 inhibitors, tumor necrosis factor‐α

## Abstract

**Background:**

Sodium‐glucose co‐transporter 2 (SGLT2) inhibitors have shown promise in reducing atrial fibrillation (AF) risk, but their mechanism of action in metabolic syndrome (MetS)‐related AF remains unclear. This study aims to elucidate the potential mechanisms by which SGLT2 inhibitors interfere with AF initiation in MetS through comprehensive bioinformatic analysis.

**Methods:**

A network pharmacology bioinformatic approach was employed to predict the molecular targets of SGLT2 inhibitors. Public databases, including SwissTarget, GeneCards, OMIM, and STRING, were utilized to identify and analyze targets associated with MetS‐related AF. Protein–protein interaction (PPI) networks were constructed, followed by gene ontology (GO) and KEGG pathway enrichment analyses to elucidate the biological processes and pathways involved.

**Results:**

A total of 52 common targets were identified linking SGLT2 inhibitors with MetS‐related AF. The analysis highlighted the TNF‐α and AGE‐RAGE signaling pathways as key mechanisms through which SGLT2 inhibitors may mitigate AF. Our investigation identified p38 and JNK, components of the TNF‐α signaling pathway, as primary targets in reducing atrial remodeling and fibrosis.

**Conclusions:**

This study provides insights into the potential mechanisms by which SGLT2 inhibitors influence AF pathogenesis in MetS. The findings suggest that targeting the TNF‐α signaling pathway may be a promising therapeutic strategy, with further experimental validation needed to confirm these results.

**Trial Registration:** The authors have nothing to report.

## Graphical abstract

Sodium‐glucose co‐transporter 2 (SGLT2) inhibitors may prevent atrial fibrillation (AF) in patients with metabolic syndrome by targeting the tumor necrosis factor‐alpha (TNF‐α) signaling pathway. This bioinformatic study suggests that SGLT2 inhibitors act on the p38 and JNK components of the TNF‐α pathway to prevent electrical and structural remodeling in AF development.

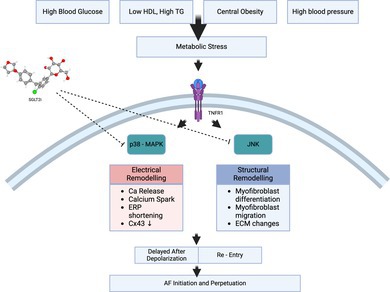

## 1 Introduction

Atrial fibrillation (AF) is a cardiac rhythm disturbance characterized by chaotic atrial activation, causing the atria to fibrillate. The global prevalence of AF is ~60 million cases, making it the most prevalent type of arrhythmia [1]. This condition is associated with several significant clinical consequences, including cardiovascular death, stroke, and heart failure [2]. Despite substantial advancements in understanding the pathophysiology and management of AF, several questions remain unanswered for physicians and researchers.

According to the latest guidelines, one of the key components of AF management is addressing cardiovascular risk factors. In the “AF‐CARE” management pathway for AF, the first “C” stands for cardiovascular risk factor management [3]. Managing diabetes mellitus (DM), obesity, hypertension, and other cardiovascular risk factors has been shown to be beneficial in both the primary and secondary prevention of AF [4]. These risk factors—hypertension, obesity, diabetes mellitus, and dyslipidemia—are collectively classified as metabolic syndrome (MetS). MetS is another prevalent health issue, with some studies reporting a prevalence of 3%–5% in the general population [5]. Each component of MetS independently increases the risk of developing AF; however, when combined as a single clinical entity, patients with MetS have a 1.57 times higher risk of developing AF compared to those without MetS [6].

Sodium‐glucose co‐transporter 2 (SGLT2) inhibitors are at the center stage of cardiovascular drug research, especially because of their potential effect in various cardiac conditions. Initially developed as glucose‐lowering agents for treating type 2 diabetes mellitus (T2DM), they are now being investigated for their therapeutic potential in a range of associated comorbidities, including chronic kidney disease and heart failure [7, 8]. SGLT2 inhibitors have demonstrated significant potential in improving cardiovascular outcomes and have also shown promise in reducing the risk of various arrhythmias. A meta‐analysis of 20 randomized controlled trials (RCTs) involving 63 604 patients found that the administration of SGLT2 inhibitors (dapagliflozin, empagliflozin, canagliflozin, and ertugliflozin) to standard therapy significantly reduced the risk of AF in patients with T2DM, heart failure, and chronic kidney disease [9]. Another study highlighted the benefits of SGLT2 inhibitors in patients with MetS, showing reductions in blood glucose levels, systolic blood pressure, and waist circumference [10].

**FIGURE 1:**
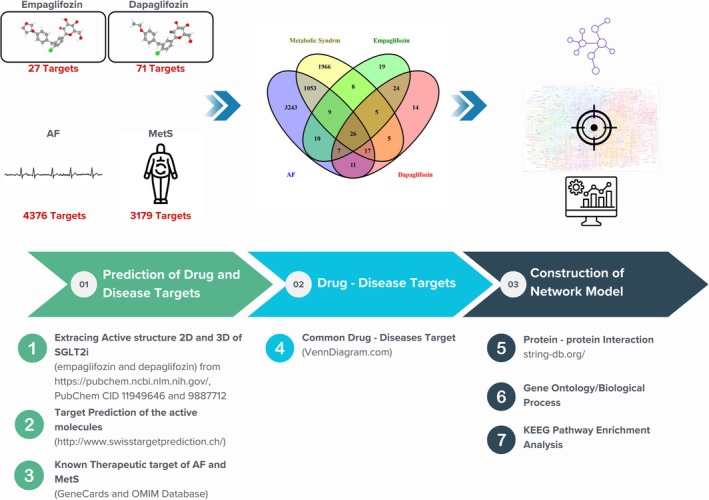
Study Workflow.

However, the precise mechanism by which SGLT2 inhibitors interfere with the initiation of AF in MetS is not yet fully understood. Elucidating the mode of action (MoA) of SGLT2 inhibitors is challenging, given the complexity of both MetS and AF. In this study, we employed bioinformatic analysis to identify therapeutic targets, molecular processes, and pathways involved. Bioinformatics integrates knowledge of biological processes with statistics and computer science and has recently proven useful in uncovering the MoA of specific drugs [11]. The process involves obtaining disease‐ or drug‐related data from databases, performing pathway enrichment analysis, and analyzing protein–protein interactions collectively referred to as network pharmacology [12]. Therefore, the study aims to investigate the potential mechanisms by which SGLT2 inhibitors may interfere with the initiation of AF in patients with MetS through a systematic bioinformatic analysis. The overall study flow diagram is depicted in Figure 1.

## 2 Materials and Methods

### 2.1 Prediction of Empagliflozin and Dapagliflozin‐Related Targets

Among the five SGLT2 inhibitors currently approved on the market, this study focused on empagliflozin and dapagliflozin due to their widespread availability. The chemical structures of empagliflozin and dapagliflozin were retrieved from PubChem (https://pubchem.ncbi.nlm.nih.gov/, PubChem CID 11949646 and 9 887 712, respectively) [13]. Target prediction was then conducted using SwissTargetPrediction (http://www.swisstargetprediction.ch/) and DrugBank (https://www.drugbank.ca/), which utilize two‐dimensional and three‐dimensional similarity measures [14, 15]. By inputting the chemical structures into these platforms, potential targets for empagliflozin and dapagliflozin were forecasted. Our selection of SwissTargetPrediction is justified by its established reliability and high accuracy, validated in numerous cheminformatics studies for predicting protein targets of small molecules [14]. The predicted targets were standardized using the Universal Protein Resource (UniProt, http://www.uniprot.org/) for further analysis [16].

### 2.2 Prediction of Known Therapeutic Targets in Metabolic Syndrome and Atrial Fibrillation

To identify target genes associated with MetS and AF, the GeneCards and OMIM databases were queried using the keywords “metabolic syndrome,” “metabolic syndromes,” and “atrial fibrillation.” GeneCards was chosen as a foundational resource for its comprehensive and manually curated data aggregated from over 150 databases, making it a robust tool for identifying disease‐related gene sets [17]. GeneCards contains data on over 7000 human genes with approved gene symbols [17], while OMIM serves as a comprehensive resource for human genes and hereditary diseases [18]. These databases provided valuable references for identifying disease‐related targets. We compared the predicted targets of SGLT2 inhibitors with disease‐related targets and used Microsoft Office Excel 2016 to screen for overlapping targets using a semi‐automated process to minimize error. The intersection of these targets (common targets) was then visualized using a Venn diagram generated on Venny 2.1 (https://bioinfogp.cnb.csic.es/tools/venny/) [19].

### 2.3 Construction of the Network Model

Empagliflozin and dapagliflozin, along with their predicted targets and the common targets of MetS and AF, were imported into Cytoscape v3.6.1 to construct the following networks: [1] a network representing the relationship between empagliflozin, dapagliflozin, and their respective targets; and [2] a network illustrating the connections between the two SGLT2 inhibitors, their common targets, and the associated disorders, namely MetS and AF. Cytoscape is a software utilized to visualize complex interaction networks between proteins, DNA, and genes [20].

### 2.4 Core Targets of Empagliflozin and Dapagliflozin in the Treatment of AF With MetS

The STRING database (https://string‐db.org) was used to analyze protein–protein interactions (PPI). This database incorporates data from co‐expression studies, biological literature, high‐throughput experiments, and genomic contexts, making it one of the most comprehensive datasets for PPI [21]. “Multiple proteins” and “ *Homo sapiens* ” as the organism of interest were selected. The PPI network was generated by incorporating the common targets of SGLT2 inhibitors with the combined MetS and AF status. A high confidence score threshold of 0.90 was set to ensure high‐quality data and minimize false positives, as a score of 0.90 or greater in the STRING database corresponds to a very low false discovery rate (FDR). Disconnected proteins were excluded from the analysis. The PPI network data were exported in “TSV” format and reconstructed using Cytoscape v3.6.1 for the topological analysis of the network. The Cytohubba plugin in Cytoscape was employed to calculate the “Degree” metric, identifying the primary targets common to both drugs and diseases. We selected the top 50% of proteins based on their high degree values, which are indicative of a protein's central and critical role in the network. This approach allowed for the identification of core targets [22].

### 2.5 Building a PPI Network for Core Targets

The PPI network for core targets was constructed using the STRING web platform. After selecting the “Multiple Protein” module, a Gene Name list of target proteins was uploaded, and the analysis was confined to proteins from *Homo sapiens* , with a confidence score set above 0.9. In the PPI diagram, each solid circle represents a gene, with the center symbolizing the protein's structure. Interconnected by lines of varying colors, the circles represent a biological process, such as gene expression regulation, signal transduction, or cell migration.

### 2.6 GO Enrichment Analysis and KEGG Pathway Enrichment Analysis

GO (gene ontology) and KEGG (Kyoto Encyclopedia of Genes and Genomes) pathway enrichment analyses were conducted using the ShinyGO 0.8 platform (http://bioinformatics.sdstate.edu/go/). These analyses were aimed at gaining a deeper understanding of the biological processes associated with core genes. For statistical significance, we applied a false discovery rate (FDR) correction with a *p* value cutoff of < 0.05. ShinyGO, built with multiple R/Bioconductor programs, provides a comprehensive annotation and pathway database from various sources [23].

## 3 Results

### 3.1 Potential Targets of Empagliflozin and Dapagliflozin

**FIGURE 2:**
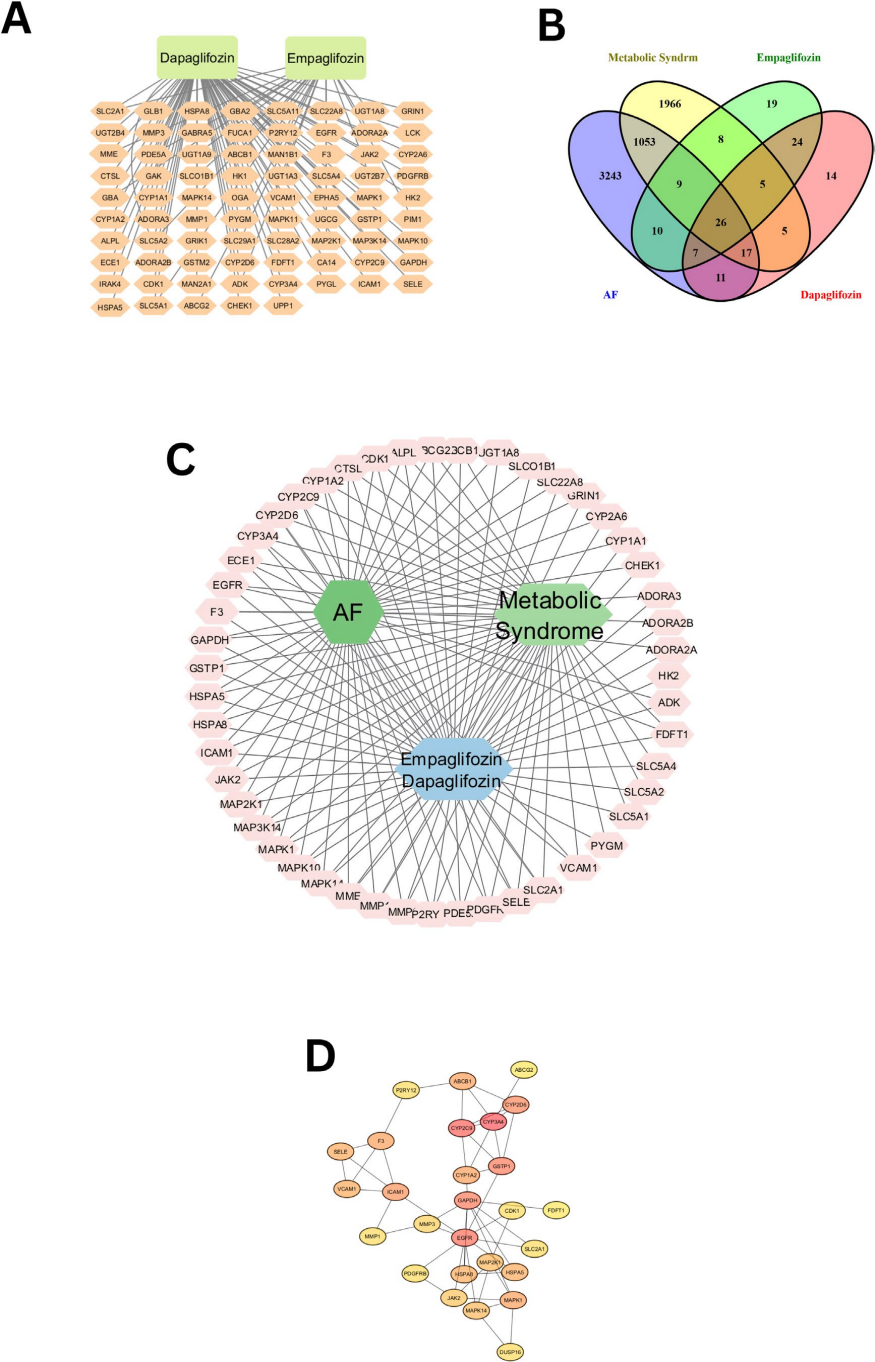
Drug‐target relationship between empagliflozin and dapagliflozin, followed by intersecting targets and core targets identification. (a) Interaction network illustrating the drug‐target relationships for Dapagliflozin and Empagliflozin; (b) Venn diagram showing the overlapping targets for empagliflozin, dapagliflozin, and targets related to AF and MetS; (c) Drug‐target‐disease network for empagliflozin and dapagliflozin in the context of AF and MetS. Blue nodes represent SGLT2 inhibitors, green nodes represent AF and MetS, and pink nodes represent the 52 common targets; (d) Core targets identified using Cytohubba, ranked by degree value. From the 52 common targets, 27 core targets were selected based on their high degree values.

A total of 98 targets with a probability score > 0 were retrieved from the public databases. Of these, 71 targets were associated with dapagliflozin, and 27 were linked to empagliflozin. After merging the data and eliminating duplicates, a total of 77 potential targets were identified for SGLT2 inhibitors. These targets were then analyzed using Cytoscape to visualize the drug‐target interaction network (Figure 2A). The network contains 79 nodes, representing 77 targets and the two drugs, empagliflozin and dapagliflozin, connected by 95 edges. In this figure, the green nodes correspond to the SGLT2 inhibitors, and the pink nodes represent the predicted drug targets. The edges illustrate the interactions between the drugs and their targets, offering insight into the potential ways in which SGLT2 inhibitors interact with multiple targets.

### 3.2 Analysis and Construction of Drug‐Target‐Disease Network

After deduplication, a combined total of 4376 targets associated with AF and 3179 targets linked to MetS from the GeneCards and OMIM databases were identified. Furthermore, 52 targets that were common/overlapping to the predicted SGLT2 inhibitor targets, AF‐related targets, and MetS‐related targets were identified (Figure 2B). The 52 overlapping targets were then used to construct a drug‐target‐disease network in Cytoscape (Figure 2C). This network consisted of 56 nodes—52 targets, two drugs, and two diseases—connected by 130 edges. In this network, blue nodes corresponded to the SGLT2 inhibitors (dapagliflozin and empagliflozin), green nodes to AF and MetS, and pink nodes symbolized the 52 common targets. The analysis revealed a strong correlation between the SGLT2 inhibitors and multiple targets in the management of AF with MetS. Key targets identified include CYP2C9, CYP2A6, CYP1A1, and CYP3A4, enzymes known for their role in metabolizing various drugs, which may interact with SGLT2 inhibitors in the treatment of these conditions. These targets provide a connection between SGLT2 inhibitors and diseases, offering a more reliable basis for investigating the mechanism of SGLT2 inhibitors in treating AF with MetS.

### 3.3 Protein–Protein Interaction Network (PPI) Core Target Analysis

**FIGURE 3:**
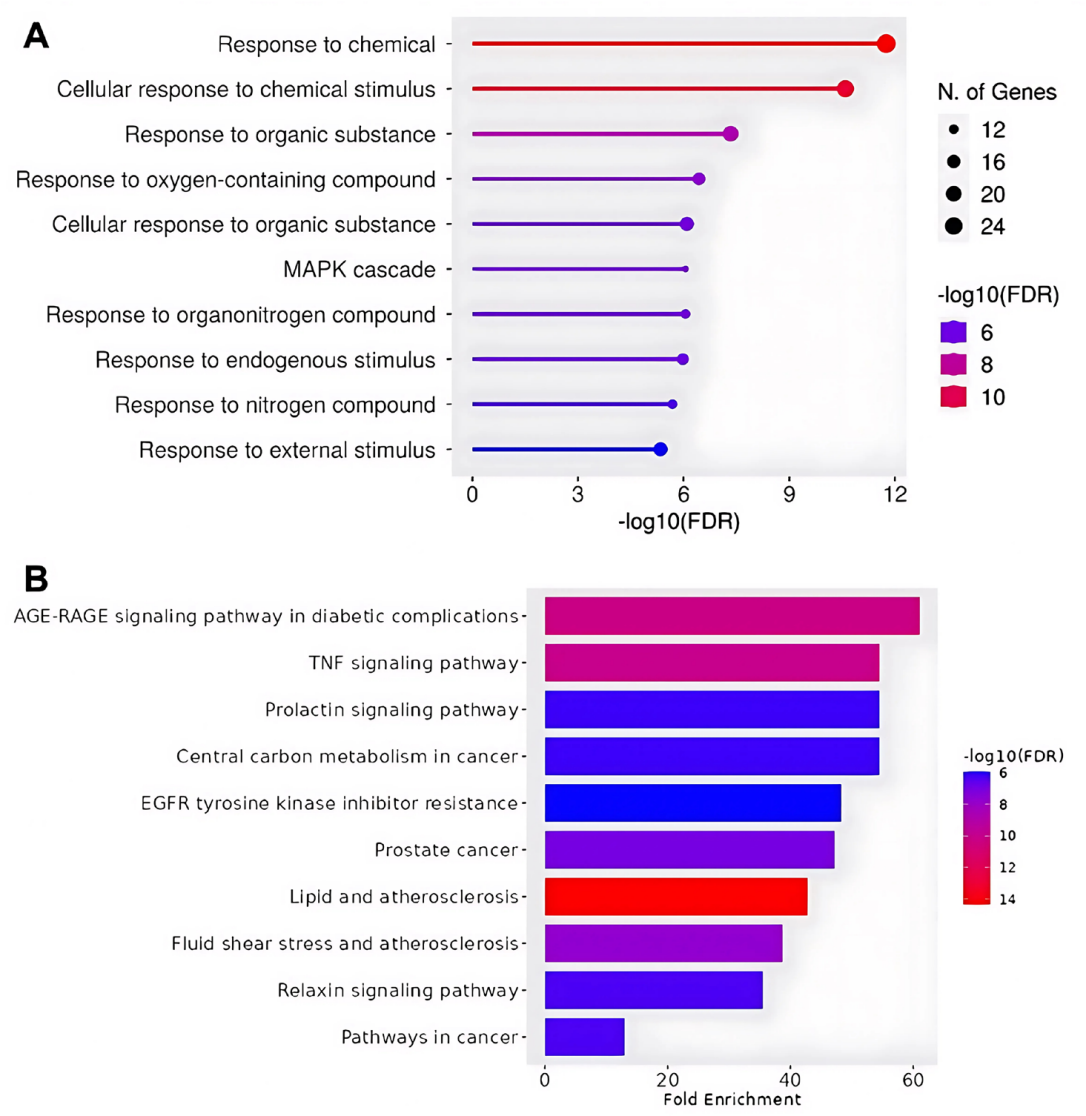
GO annotations and KEGG pathway enrichment analysis of SGLT2 inhibitors core targets in AF with MetS. (a) Enrichment analysis of gene ontology (GO) biological processes related to empagliflozin and dapagliflozin in atrial fibrillation with metabolic syndrome; (b) KEGG pathway enrichment analysis of core gene targets for empagliflozin and dapagliflozin in atrial fibrillation with metabolic syndrome.

To thoroughly explore the effects of empagliflozin and dapagliflozin on AF in individuals with MetS, we conducted a detailed analysis using the R statistical programming language. Our focus was on the 27 core target genes common to both SGLT2 inhibitors and the intersection of AF and metabolic syndrome. Using the GO and KEGG pathway databases, we analyzed the biological functions and signaling pathways associated with these genes. We applied filters with a *p* value and *Q* value threshold < 0.05, resulting in the identification of 10 significant biological processes from the 33 core targets (Figure 3A). These processes include responses to chemicals, cellular responses to chemical stimuli, responses to organic substances, responses to oxygen‐containing compounds, cellular responses to organic substances, MAPK cascades, responses to organonitrogen compounds, responses to endogenous stimuli, responses to nitrogen compounds, and responses to external stimuli.

Based on the KEGG enrichment analysis of the 27 key targets, we identified the top 10 signaling pathways with high confidence (*P* value < 0.05) (Figure 3B). The results suggest that the core targets may influence pathways such as the AGE‐RAGE signaling pathway in diabetic complications, TNF signaling pathway, prolactin signaling pathway, and EGFR tyrosine kinase inhibitor resistance. These findings predict that empagliflozin and dapagliflozin could be effective in treating AF with metabolic syndrome by modulating these specific signaling pathways.

## 4 Discussion

There are potentially two gaps in evidence that need to be cleared in the role of SGLT2 inhibitors as antiarrhythmic agents, especially in AF‐related MetS. Lack of large RCT and lack of precise biomolecular pathways. Bioinformatics analysis approaches had potential roles for finding those pathways by analyzing complex biological data, looking for new potential targets, and finding drug‐disease interactions. From our final analysis, we identified 52 common and 27 core targets associated with metabolic syndrome‐related atrial fibrillation (AF) that are influenced by SGLT2 inhibitors. Based on the quantitative metric of “Degree” from the PPI network analysis, we identified MAPK14 (p38) and MAPK8 (JNK) as key core targets. These findings are validated by existing literature, which has shown their involvement in inflammatory signaling and their role as targets for other cardiovascular drugs, providing strong biological plausibility.

These targets belong to several biological pathways. Among these, two critical pathways—TNF (tumor necrosis factor) signaling and AGE‐RAGE (advanced glycation end‐product‐receptor for AGE) signaling—were identified as the most significant mechanisms by which SGLT2 inhibitors exert their effects in patients with MetS‐related AF. As shown in Figure 3B, both pathways showed comparably high fold enrichment scores, indicating that SGLT2 inhibitors may act on a combination of these pathways to achieve their therapeutic effects.

The AGE‐RAGE pathway had already been known as a major signaling pathway related to diabetes complication [24]. The accumulation of AGEs caused by chronic hyperglycemia in diabetes patients activates the RAGE receptor, leading to the release of pro‐inflammatory cytokines like TNF‐α and inducing oxidative stress. This process is a major contributor to vascular dysfunction and tissue remodeling, which are central to both MetS and AF pathogenesis [25]. As previously mentioned, SGLT2 inhibitors act primarily as glucose‐lowering drugs; thereby, inhibition in this pathway is very well understood.

TNF‐α, a member of the larger TNF family of structurally related proteins, plays a crucial role in various biological processes, including inflammation, tumor suppression, and viral replication. The term TNF was first introduced in 1975 to describe a cytotoxic factor produced by macrophages, capable of killing fibrosarcoma cells in rats. TNF‐α is encoded by a 3‐kb gene located on chromosome 6p21.3 [26].

TNF is a pro‐inflammatory cytokine produced by adipose tissue and is a key factor in the development of metabolic inflammation, or meta‐inflammation, which is also pivotal in metabolic syndrome pathogenesis [27]. TNF‐α, also known as TNF superfamily member 2 (TNFSF2), is a multifunctional cytokine involved in both innate and adaptive immune responses. Under normal conditions, TNF‐α production is tightly regulated and time‐limited. However, in chronic inflammatory diseases, there is sustained production of TNF‐α. Patients with hypertension, type 2 diabetes mellitus (DMT2), and dyslipidemia—all components of MetS—experience a state of low‐grade chronic inflammation. Elevated levels of angiotensin II (ANGII) in hypertensive patients stimulate the production of TNF‐α, which in turn causes vasoconstriction and hypoperfusion of the kidneys [28].

AGE‐RAGE and TNF‐α signaling pathways are not independent pathways but part of an interconnected network. The activation of RAGE by AGEs can be defined as an upstream event that can lead to the production of TNF‐α. Therefore, SGLT2 inhibitors may exert their effects through a dual mechanism: first, by reducing blood glucose that drives AGE formation, and second, by modulating the TNF‐α pathway directly and indirectly. This combined action provides a stronger biological rationale for the efficacy of SGLT2 inhibitors in MetS‐related AF.

The initiation and perpetuation of AF are closely linked to atrial electrical and structural remodeling [29]. The same pathogenic process is believed to connect MetS‐related AF, with the TNF signaling pathway playing a significant role in promoting structural remodeling. Crosstalk between ANGII and TNF‐α promotes atrial and ventricular fibrosis by upregulating profibrotic gene expression, including collagen I, collagen III, and connective tissue growth factor (CTGF) [30]. Excessive expression of TNF‐α can lead to apoptosis of cardiac myocytes through both extrinsic and intrinsic pathways [31]. Moreover, in mice model, overexpression of TNF‐α was associated with atrial hypertrophy and enlargement, though the precise mechanisms remain unclear. Other observed effects include increased collagen accumulation, altered connexin‐40 expression via the Smad pathway, activation of myofibroblasts, and reduced matrix metalloproteinase (MMP) secretion in the atrial wall of the TNF‐α model mice compared to the control [32].

Substantial evidence suggests that TNF‐α plays a critical role in electrical remodeling, particularly in calcium homeostasis through its influence on NCX, calcium channels, and SERCA. Additionally, remodeling of gap junctions due to connexin‐43 abnormalities contributes to the development of AF [33]. The inhibition of noradrenaline release by TNF‐α also indicates the presence of autonomic dysfunction.

**FIGURE 4:**
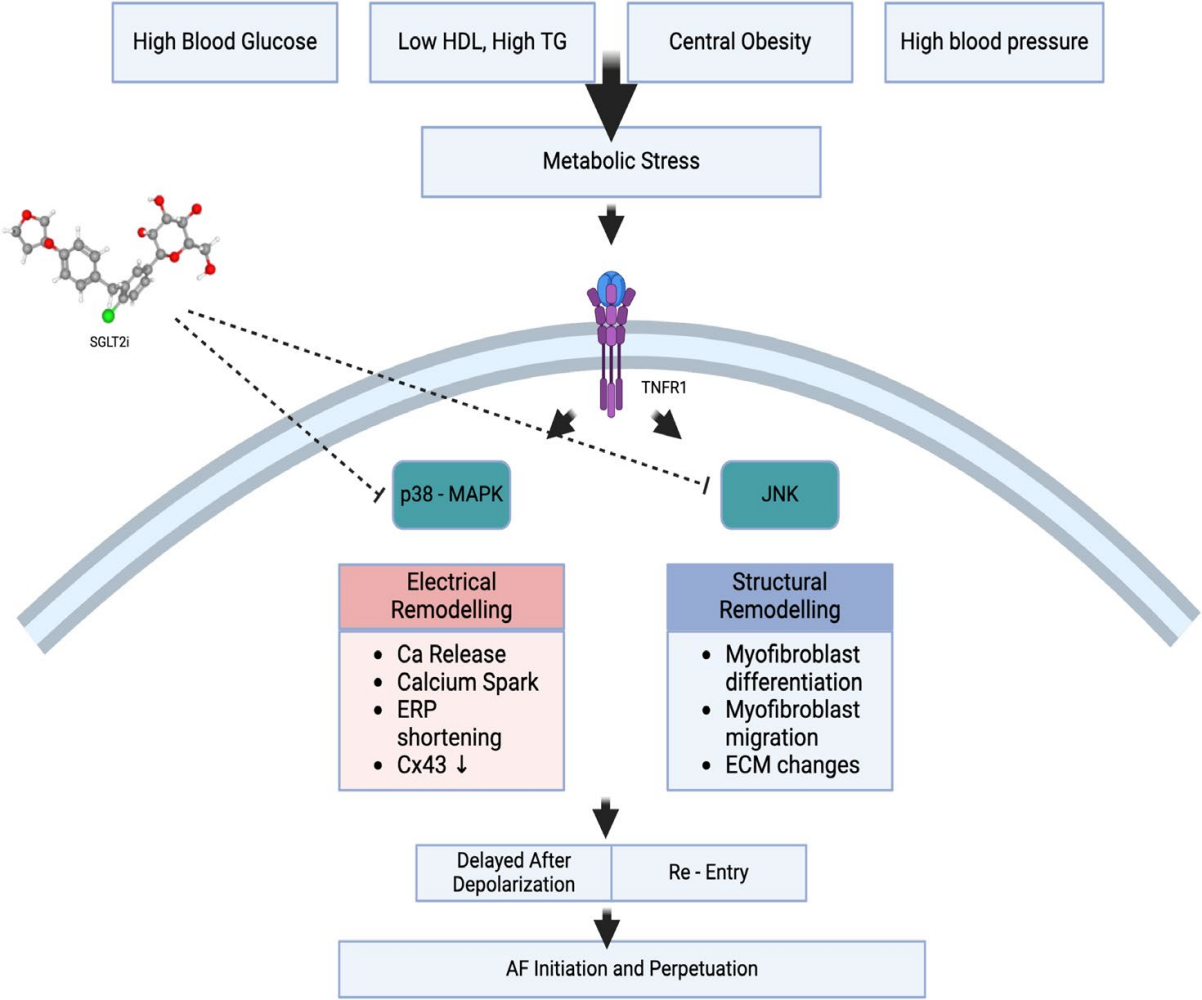
Final proposed molecular mechanism of SGLT2i in AF‐related metabolic syndrome (created with Biorender.com).

Network analysis and protein—protein Interaction investigation revealed that p38 and JNK are the primary targets of SGLT2 inhibitors in mitigating AF associated with MetS (Figure 4). These two pathways are key downstream components of the TNF‐α signaling cascade and belong to the mitogen‐activated protein kinase (MAPK) family. MAPKs are a group of signaling pathways that translate extracellular stress stimuli into a wide range of intracellular responses [34]. While many signaling pathways are part of this group, three major pathways have been identified in mammals: the extracellular signal‐regulated kinase 1/2 (ERK1/2), the Jun N‐terminal kinase (JNK), and the p38 pathway. The ERK1/2 pathway is primarily involved in tumorigenesis, while the JNK and p38 pathways play more significant roles in responding to environmental stress, such as chronic inflammation [35]. By targeting these two major pathways involved in TNF‐α signaling, the downstream processes can be effectively blocked. This blockade is expected to minimize the electrical and structural remodeling of atrial myocytes, which is a key factor in the initiation and perpetuation of AF in patients with MetS.

Network pharmacology offers a novel approach to exploring the complex interactions between drugs and disease conditions. In our study, this method helped identify potential molecular targets of SGLT2 inhibitors, particularly in relation to metabolic syndrome‐related atrial fibrillation. Through this systematic bioinformatic analysis, we identified key enrichment pathways associated with these targets and proposed potential therapeutic targets for SGLT2 inhibitors in the context of AF with MetS. However, a major limitation of the study is the lack of experimental validation. Therefore, further pharmacological studies are essential to clarify the exact relationship between SGLT2 inhibitors and AF with MetS. Future research should also focus on validating our findings at the molecular level to confirm the proposed mechanisms and therapeutic implications.

## 5 Conclusions

Taken together, our study systematically predicted, screened, and analyzed the targets and pathways that might play a vital role in the biological process, which elaborated the possible mechanisms of SGLT2 inhibitors in MetS‐related AF. Most importantly, these results provide evidence and new insights for further research on the pharmacological mechanism of SGLT2 inhibitors in targeting the TNF‐α signaling pathway as the key pathogenesis of AF in MetS.

## Author Contributions

A.R., M.S.R., F.F., H.S., Ah.R., and S.P.: conceptualization. A.R., A.P., and M.S.R.: methodology. A.R., A.P.: software, writing – original draft preparation. M.S.R., F.F., H.S., Ah.R., and S.P.: writing – review and editing. M.S.R., F.F., and H.S.: supervision. All authors have read and agreed to the published version of the manuscript.

## Disclosure

Permission to Reproduce Material From Other Sources: Image was generated with licensed Biorender.com apps.

## Ethics Statement

The authors have nothing to report.

## Consent

The authors have nothing to report.

## Conflicts of Interest

The authors declare no conflicts of interest.

## Data Availability

The datasets generated during this study are available from the corresponding author on reasonable request.
